# Spin Hall Effect in the Paraxial Light Beams with Multiple Polarization Singularities

**DOI:** 10.3390/mi14040777

**Published:** 2023-03-30

**Authors:** Alexey A. Kovalev, Victor V. Kotlyar, Sergey S. Stafeev

**Affiliations:** 1 Image Processing Systems Institute of the RAS—Branch of FSRC “Crystallography & Photonics” of the RAS, 151 Molodogvardeyskaya St., 443001 Samara, Russia; 2 Samara National Research University, 34 Moskovskoe Shosse, 443086 Samara, Russia

**Keywords:** cylindrical vector beam, radial polarization, polarization singularity, Gaussian beam, Laguerre–Gaussian beam, spin angular momentum, optical spin Hall effect, orbital angular momentum

## Abstract

Elements of micromachines can be driven by light, including structured light with phase and/or polarization singularities. We investigate a paraxial vectorial Gaussian beam with multiple polarization singularities residing on a circle. Such a beam is a superposition of a cylindrically polarized Laguerre–Gaussian beam with a linearly polarized Gaussian beam. We demonstrate that, despite linear polarization in the initial plane, on propagation in space, alternating areas are generated with a spin angular momentum (SAM) density of opposite sign, that manifest about the spin Hall effect. We derive that in each transverse plane, maximal SAM magnitude is on a certain-radius circle. We obtain an approximate expression for the distance to the transverse plane with the maximal SAM density. Besides, we define the singularities circle radius, for which the achievable SAM density is maximal. It turns out that in this case the energies of the Laguerre–Gaussian and of the Gaussian beams are equal. We obtain an expression for the orbital angular momentum density and find that it is equal to the SAM density, multiplied by −*m*/2 with *m* being the order of the Laguerre–Gaussian beam, equal to the number of the polarization singularities. We consider an analogy with plane waves and find that the spin Hall affect arises due to the different divergence between the linearly polarized Gaussian beam and cylindrically polarized Laguerre–Gaussian beam. Application areas of the obtained results are designing micromachines with optically driven elements.

## 1. Introduction

In micromachines, elements that can be driven by light and optical vortex beams are widely adopted in optical tweezers. One of the natural generalizations of optical vortex beams are light fields with multiple vortices. Rather general expressions for describing such fields have been obtained by G. Indebetouw [1] and E.G. Abramochkin et al. [2]. Such fields propagate in free space without changing their transverse intensity shape, up to scale and rotation around the optical axis. In addition to the optical vortices, which are phase singularities or points with an uncertain phase, vector light fields can have polarization singularities, where uncertain is polarization. Polarization singularities were investigated in a huge number of papers. Recently, a review about polarization singularities was published [3]. Most widely studied polarization singularities are radial and azimuthal polarization, and their superposition known as cylindrical polarization [4]. Such light fields can be constructed as superpositions of optical vortices with opposite circular polarizations and with opposite topological charges of ±1 [5]. Based on this scheme, we investigated in 2018 light fields with multiple polarization singularities residing on a circle with the center on the optical axis [6,7]. Such fields have locally linear polarization. We found that, in contrast to the phase singularities, which conserve in an arbitrary transverse plane, polarization singularities appear only in a discrete number of transverse planes. We discovered that the singularities can transform, for instance, from radial and azimuthal polarization in the initial plane to, respectively, azimuthal and radial polarization in the far field. However, we did not investigate what happens with polarization between the planes where polarization singularities reconstructed, i.e., between the planes with locally linear polarization. In [8], it was noticed that along the propagation direction, such singularities generically split into a pair of C-points with opposite circular polarization. As it turns out, such splitting is a manifestation of the optical spin Hall effect, which means that alternating areas with the spin angular momentum of opposite sign appear, despite linear polarization in the initial plane. The Hall effect was discovered for metals by Edwin Hall back in 1878. Later, in 1971, it was discovered by M.I. Dyakonov and V.I. Perel for semiconductors [9]. In 2004, this effect was also found to occur in photonics [10]. The photonic spin Hall effect [11,12] consists of the separation of photon paths depending on their spin. The optical Hall effect can be divided into a spin Hall effect [13], orbital Hall effect [14], and spin-orbital Hall effect [15]. The spin Hall effect can take place when light is passed through a medium interface [16] or an inhomogeneous medium or in microcavities [17], metamaterials [18], dielectric gratings [19], and in free space in the tight focusing conditions [20].

Thus, we were interested in whether such an effect can arise in free space, without any material structures, and even without tight focusing, i.e., in paraxial light beams, that can be easily generated by a spatial light modulator.

In this work, therefore, we investigate this phenomenon in the paraxial Gaussian beam with multiple polarization singularities from [6,7]. Such a beam is a superposition of a cylindrically polarized Laguerre–Gaussian (LG) beam with a linearly polarized Gaussian beam. We found that maximal SAM density magnitudes appear on a circle and the radius of this circle has been derived. It was obtained that the maximal SAM density is generated in a certain transverse plane, the distance to which has been obtain approximately. It was derived that the maximal SAM density can be achieved when the singularities circle has a definite radius, such that the energy of the Gaussian beam is equal to the energy of the LG beam. We revealed the mechanism of the spin Hall effect in such beams. This effect arises due to the different divergence of the LG beam and of the Gaussian beam.

## 2. Paraxial Light Fields with Multiple Phase or Polarization Singularities

It has been shown (Equation (17) in [2]) that any function given by

(1) $$E\left(r,\varphi ,z\right)=\frac{1}{q}\exp\left(-\frac{r^{2}}{qw_{0}^{2}}\right)f\left(\frac{re^{i\varphi }}{qw_{0}}\right),$$

where (*r*, *φ*, *z*) are the cylindrical coordinates, *w*_0_ is the waist radius of the Gaussian beam, *q* = 1 + *iz*/*z*_0_, *z*_0_ = *kw*_0_^2^/2 is the Rayleigh distance, *k* is the wavenumber, and *f*(*re^iφ^*) is an arbitrary entire analytical function, describes a solution of a paraxial Schrödinger-type Helmholtz equation: 2*ik*(∂*E*/∂*z*) + (∂^2^*E*/∂*x*^2^) + (∂^2^*E*/∂*y*^2^) = 0.

The light field described by Equation (1) propagates in free space without changing its shape. The parameter *q* defines the scaling and rotation of the light field: at distance *z* the field becomes |*q*| = [1 + (*z*/*z*_0_)^2^]^1/2^ times wider and rotates around the optical axis by an angle equal to the Gouy phase *ψ* = arg *q* = arctan(*z*/*z*_0_).

Using Equation (1), it is possible to obtain a solution of the paraxial Helmholtz equation with optical vortices located in arbitrary points with their polar coordinates in the initial plane (*r_p_*, *φ_p_*) (*p* = 0, 1, …, *m* − 1). In an arbitrary transverse plane, the complex amplitude of such a field takes the form [1]:

(2) $$E\left(r,\varphi ,z\right)=\frac{1}{qw_{0}^{m}}\exp\left(-\frac{r^{2}}{qw_{0}^{2}}\right)\prod_{p=0}^{m-1}\left(\frac{re^{i\varphi }}{q}-r_{p}e^{i\varphi_{p}}\right).$$

Choosing the vortices on a circle with the radius *a*_0_, i.e., *r_p_* = *a*_0_, *φ_p_* = 2π*p*/*m*, we get

(3) $$E\left(r,\varphi ,z\right)=\frac{1}{qw_{0}^{\left|m\right|}}\exp\left(-\frac{r^{2}}{qw_{0}^{2}}\right)\left[{\left(\frac{r}{q}\right)}^{\left|m\right|}e^{im\varphi }-a_{0}^{\left|m\right|}\right].$$

This field is a superposition of a single-ringed *m*th-order Laguerre–Gaussian (LG) beam with a fundamental Gaussian beam.

It has been known [5] that radially polarized light can be expressed as a superposition of optical vortices of the ±1st order and of the opposite handedness since the Jones vector can be presented as

(4) $$\left(\begin{array}{c} \cos\varphi \\ \sin\varphi \end{array}\right)=\frac{1}{2}e^{i\varphi }\left(\begin{array}{c} 1\\ -i \end{array}\right)+\frac{1}{2}e^{-i\varphi }\left(\begin{array}{c} 1\\ i \end{array}\right).$$

If, in this expression, *e^iφ^* and *e*^–*iφ*^ are replaced by the field (3) of the orders, respectively, *m* and −*m*, we get a vector light field with *m* polarization singularities located on a circle of the radius *a*_0_ [6,7]:

(5) $$E\left(r,\varphi ,z\right)=\frac{1}{q^{m+1}w_{0}^{m}\sqrt{W_{0}}}\exp\left(-\frac{r^{2}}{qw_{0}^{2}}\right)\left[\begin{array}{l} r^{m}\cos m\varphi -a_{0}^{m}q^{m}\\ r^{m}\sin m\varphi \end{array}\right],$$

where the multiplier

(6) $$W_{0}=\frac{\pi w_{0}^{2}}{2}\left[\frac{m!}{2^{m}}+{\left(\frac{a_{0}}{w_{0}}\right)}^{2m}\right]$$

is introduced for normalizing the beam energy (making it equal to unit and thus equal for all values *m* and *a*_0_).

**Figure 1:**
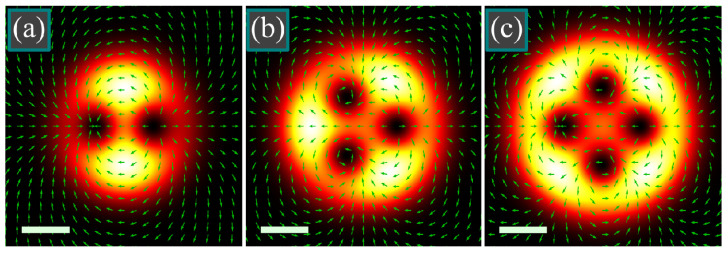
Intensity distributions of the light field (5) in the initial plane (*z* = 0) for the following parameters: wavelength λ = 532 nm, Gaussian beam waist radius *w*_0_ = 1 mm, number of polarization singularities *m* = 2 (**a**), *m* = 3 (**b**) and *m* = 4 (**c**), radius of the singularities circle *a*_0_ = 600 μm (**a**), *a*_0_ = 700 μm (**b**), *a*_0_ = 800 μm (**c**). Size of all figures is 5 × 5 mm^2^, scale mark in each figure denotes 1 mm. Arrows show the directions of linear polarization.

Examples of such field with *m* = 2, 3, 4 are shown in Figure 1. Since the vortices reside on a circle with the radius *a*_0_, from now on, we call this parameter a singularities circle radius.

We note that the field (5) can be treated as a superposition of two single-ringed Laguerre–Gaussian beams with opposite topological charges and with circular polarizations, and of a linearly polarized Gaussian beam:

(7) $$\begin{array}{l} E\left(r,\varphi ,z\right)=\frac{1}{2\sqrt{W_{0}}}{\mathrm{LG}}_{m}\left(r,\varphi ,z\right)\left[\begin{array}{c} 1\\ -i \end{array}\right]+\frac{1}{2\sqrt{W_{0}}}{\mathrm{LG}}_{-m}\left(r,\varphi ,z\right)\left[\begin{array}{c} 1\\ i \end{array}\right]\\ \;\;\;\;-\frac{1}{\sqrt{W_{0}}}{\left(\frac{a_{0}}{w_{0}}\right)}^{m}{\mathrm{LG}}_{0}\left(r,\varphi ,z\right)\left[\begin{array}{c} 1\\ 0 \end{array}\right], \end{array}$$

with LG*_m_* (*r*, *φ*, *z*) being the scalar *m*th-order single-ringed Laguerre–Gaussian beam:

(8) $${\mathrm{LG}}_{m}\left(r,\varphi ,z\right)=\frac{1}{q}{\left(\frac{r}{qw_{0}}\right)}^{\left|m\right|}\exp\left(-\frac{r^{2}}{qw_{0}^{2}}+im\varphi \right).$$

The first two terms in Equation (7) are responsible for constructing polarization singularity (*m*-order radial polarization with the Jones vector **J** = [cos *mφ*, sin *mφ*]), while the third term splits this singularity into *m* first-order polarization singularities residing on a circle of the radius *a*_0_. The same splitting effect, but for phase singularities, was reported in [21].

## 3. Intensity Distribution

From Equation (5), the intensity distribution is given by

(9) $$\begin{array}{l} I\left(r,\varphi ,z\right)={\left|E_{x}\left(r,\varphi ,z\right)\right|}^{2}+{\left|E_{y}\left(r,\varphi ,z\right)\right|}^{2}\\ \;\;\;\;=\frac{1}{{\left|q\right|}^{2m+2}w_{0}^{2m}W_{0}}\exp\left(-\frac{2r^{2}}{{\left|q\right|}^{2}w_{0}^{2}}\right)\left[r^{2m}+a_{0}^{2m}{\left|q\right|}^{2m}-2a_{0}^{m}{\left|q\right|}^{m}r^{m}\cos\left(m\psi \right)\cos\left(m\varphi \right)\right], \end{array}$$

with *ψ* = arctan(*z*/*z*_0_) being the Gouy phase.

It is seen that the intensity nulls can appear only in a discrete set of transverse planes, where cos(*mψ*) = ±1, i.e., tan(*mψ*) = 0, which is consistent with [6,7].

In the initial plane, the intensity is

(10) $$I\left(r,\varphi ,0\right)=\frac{1}{w_{0}^{2m}W_{0}}\exp\left(-\frac{2r^{2}}{w_{0}^{2}}\right)\left[r^{2m}+a_{0}^{2m}-2a_{0}^{m}r^{m}\cos\left(m\varphi \right)\right].$$

**Figure 2:**
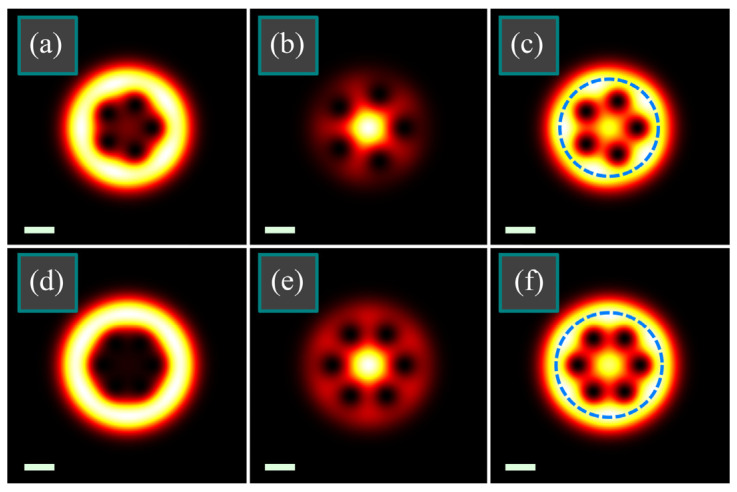
Intensity distributions of the light field (5) in the initial plane (*z* = 0) for the following parameters: wavelength λ = 532 nm, Gaussian beam waist radius *w*_0_ = 1 mm, number of polarization singularities *m* = 5 (**a**–**c**) and *m* = 6 (**d**–**f**), radius of the singularities circle *a*_0_ = 800 μm (**a**,**d**), *a*_0_ = 1200 μm (**b**,**e**), *a*_0_ = 942 μm (**c**), *a*_0_ = 1041 μm (**f**). Size of all figures is 8 × 8 mm^2^, scale mark in each figure denotes 1 mm. Blue dashed circles (**c**,**f**) denote the radius of the maximal peripheral intensity computed by Equation (20).

However, the beam from Equation (5) is a superposition of circularly polarized single-ringed LG vortex beams of the orders ±*m* and of a linearly polarized Gaussian beam. At a small singularities circle radius *a*_0_, the LG beam overwhelms and the intensity looks like a ring (Figure 2a,d). At large *a*_0_, vice versa, the Gaussian beam is brighter and the intensity looks more like a spot (Figure 2b,e). In some applications, however, it is desirable to confine the intensity nulls between the light walls. For instance, in 2008, Dienerowitz et al. showed that a vortex beam with annular profile can confine metal nanoparticles in the dark region of the beam center [22]. Thus, the intensities from Figure 2a,b,d,e are undesirable. Now, we try to find the radius *a*_0_ such that the intensity in the beam center and in the edges, beyond the intensity nulls, are nearly the same. Since the first intensity null is at *φ* = 0, this condition can be written as

(11) $$I\left(0,0,0\right)=\underset{r>a_{0}}{\max}I\left(r,0,0\right),$$

or, after taking the square root of both parts of Equation (11),

(12) $$a_{0}^{m}=\underset{r>a_{0}}{\max}\left\{\exp\left(-\frac{r^{2}}{w_{0}^{2}}\right)\left(r^{m}-a_{0}^{m}\right)\right\}.$$

Thus, we need to determine the maximal peripheral intensity, beyond the null. Taking the derivative of the right part of Equation (12) with respect to *r* yields an equation for the radial coordinate *r*_0,max_ of the maximal intensity in the initial plane:

(13) $$2\left(r_{\max}^{m}-a_{0}^{m}\right)=mw_{0}^{2}r_{0,\max}^{m-2}.$$

This equation can be solved only for small values *m*. However, we do not need to solve it, since we are interested in *a*_0_ rather than in *r*_0,max_. Expressing *a*_0_ via *r*_0,max_ and substituting it into Equation (12), we get

(14) $$r_{0,\max}^{m}-\frac{mw_{0}^{2}}{2}r_{0,\max}^{m-2}=\frac{mw_{0}^{2}}{2}r_{0,\max}^{m-2}\exp\left(-\frac{r_{0,\max}^{2}}{w_{0}^{2}}\right).$$

Division of both parts by $\left(mw_{0}^{2}/2\right)r_{0,\max}^{m-2}$ yields a simple equation:

(15) $$\frac{2}{m}\xi -1=\exp\left(-\xi \right).$$

with *ξ* = (*r*_0,max_/*w*_0_)^2^. Since for large *m* an approximate solution is *ξ* ≈ *m*/2, we denote *ξ* = *m*/2 + *η* and get

(16) $$\frac{2}{m}\;\eta \approx e^{-m/2}\left(1-\eta \right)$$

and, therefore,

(17) $$\eta \approx \frac{e^{-m/2}}{2/m+e^{-m/2}}.$$

Returning back to *ξ*, we obtain the solution

(18) $$\xi =\frac{m}{2}\left(1+\frac{1}{m/2+e^{m/2}}\right).$$

Thus, we get the singularities circle radius *a*_0_, for which the intensity in the center and at the edge (near the dark spot) is nearly the same:

(19) $$a_{0}={\left(r_{0,\max}^{m}-\frac{mw_{0}^{2}}{2}r_{0,\max}^{m-2}\right)}^{1/m},$$

with

(20) $$r_{0,\max}=w_{0}\sqrt{\frac{m}{2}}\sqrt{1+\frac{1}{m/2+e^{m/2}}}.$$

It is seen that the first two multipliers are equal to the maximal-intensity radius of a single-ringed *m*th-order LG beam with the waist radius *w*_0_, while the third multiplier tends to unit with growing number of singularities *m*. Shown in Figure 2c,f are the intensity distributions with the singularities circle radius obtained by Equation (19). These figures confirm that Equation (19) allows making the intensities in the center and in the periphery nearly equal.

In optical tweezers, the intensity distribution affects where the particles are trapped. However, the motion of particles is governed by the spin and orbital angular momenta.

## 4. Spin Angular Momentum Density

In paraxial light fields, only the longitudinal component of the SAM vector can be significant. It is equal to

(21) Sz=2ImEx*Ey,

Substituting here Equation (5) for the light field, we get

(22) $$S_{z}=\frac{2}{{\left|q\right|}^{2}W_{0}}{\left(\frac{a_{0}r}{\left|q\right|w_{0}^{2}}\right)}^{m}\exp\left(-\frac{2r^{2}}{{\left|q\right|}^{2}w_{0}^{2}}\right)\sin\left(m\psi \right)\sin\left(m\varphi \right),$$

where *ψ* = arctan(*z*/*z*_0_) is the Gouy phase.

It is seen from this expression that there are transverse planes where the SAM is zero, i.e., polarization is linear. In these planes, sin(*mψ*) = 0, i.e., they are located at the following distances [6,7]:

(23) $$z=z_{0}\tan\left(\frac{\pi p}{m}\right),$$

with *p* = 0, 1, …, [*m*/2], where [.] means the integer part of a fractional number.

In other planes, the SAM is generally nonzero, but in each plane, it equals to zero at the polar angles *φ_p_* = π*p*/*m* with *p* = 0, …, *m* − 1.

Comparison of the expressions for the SAM and for the intensity reveals that in an arbitrary transverse plane, the light field has C-points, where polarization is circular [23,24]. Equation $S_{z}\left(r,\varphi ,z\right)=\pm I\left(r,\varphi ,z\right)$ leads to the following C-points coordinates:

(24) $$\left\{\begin{array}{l} r=a_{0}\left|q\right|,\\ \varphi =\pm \psi +\frac{2\pi p}{m}, \end{array}\right.$$

where *p* = 0, …, *m* − 1. Thus, there are *m* points with right circular polarization (at *φ* = *ψ* + 2π*p*/*m*) and *m* points with left circular polarization (at *φ* = –*ψ* + 2π*p*/*m*).

**Figure 3:**
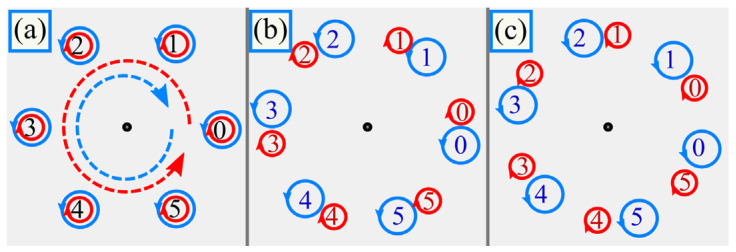
Evolution of the C-points in the light field (5). In the initial plane (**a**), polarization is linear, i.e., opposite C-points reside in the same points and compensate each other. When starting to propagate (**b**), C-points with right and left circular polarization rotate in the transverse plane in opposite directions. When approaching the next plane with linear polarization (**c**), given by Equation (23), C-points with right and left circular polarization merge again. The numbers 0–5 denote respective C-points, while red and blue color means respectively right and left circular polarization.

It is seen that on propagation, C-points with right and left circular polarization are rotated around the optical axis in opposite directions. When passing through the planes given by Equation (23), coordinates of these C-points coincide, they annihilate each other and polarization becomes linear. Evolution of the C-points is illustrated in Figure 3.

Now, we try to determine where the SAM achieves zero or maximal magnitudes.

If sin(*mψ*) > 0 in Equation (22), then the maximal and minimal SAM density is achieved, respectively, at the polar angles *φ_p_* = (π + 4π*p*)/(2*m*) and *φ_p_* = (–π + 4π*p*)/(2*m*) with *p* = 0, …, *m* − 1, and these angles are independent of the propagation distance and on the singularities circle radius *a*_0_. However, after passing the planes with linear polarization and with the polarization singularities (Equation (23)), the angles of the maximal and minimal SAM density are swapped.

Differentiating Equation (22) by *r* yields that at a fixed propagation distance *z* and at the angles *φ_p_*, maximal SAM density is achieved on a circle with the radius

(25) $$r=\frac{w_{0}\left|q\right|}{2}\sqrt{m}.$$

This radius is √2 times smaller than the radius of maximal intensity of a single-ringed *m*th-order LG beam, i.e., of the beam from Equation (8), whose maximal-intensity ring radius is *r* = *w*_0_|*q*|(*m*/2)^1/2^ (Equation (10) in [25]).

Substituting the radius from Equation (25) into Equation (22) for the SAM, we get the maximal SAM density in a transverse plane at a distance *z* from the initial plane:

(26) $$\underset{r,\varphi }{\max}S_{z}=\frac{2}{{\left|q\right|}^{2}W_{0}}{\left(\frac{a_{0}\sqrt{m}}{2w_{0}}\right)}^{m}\exp\left(-\frac{m}{2}\right)\sin\left(m\psi \right).$$

Now, after obtaining the maximal SAM in each transverse plane, we find the planes with where the SAM achieves the maximal value. For this plane, the following condition should be fulfilled:

(27) $$\frac{\partial }{\partial z}\left(\underset{r,\varphi }{\max}S_{z}\right)=0,$$

Substituting here the maximal SAM in the plane [Equation (26)], we get

(28) $$\frac{\partial }{\partial z}\left\{{\left(1+\frac{z^{2}}{z_{0}^{2}}\right)}^{-1}\sin\left[m\arctan\left(\frac{z}{z_{0}}\right)\right]\right\}=0.$$

This equation leads us to the following distance *z*_max_ to the plane with the maximal SAM:

(29) $$\tan\left(m\arctan\left(\frac{z_{\max}}{z_{0}}\right)\right)=\frac{mz_{0}}{2z_{\max}}.$$

This equation can also be written in a short form via the Gouy phase *ψ*_max_ of this plane:

(30) $$\tan\left(\psi_{\max}\right)\tan\left(m\psi_{\max}\right)=m/2,$$

Both these equations indicate that the distance to the plane with maximal SAM is independent of the radius of the singularities circle *a*_0_.

Equations (29) and (30) are valid for any value *m*, but analytically they can be solved only for small values *m*. For instance, *z* = *z*_0_/√2 at *m* = 1 and *z* = *z*_0_/√3 at *m* = 2. For larger values *m*, these equations lead to a problem of finding roots of high-order polynomials, but we try to estimate the solutions. Since

(31) $$\sin\left(m\psi_{\max}\right)=\frac{1}{\sqrt{1+1/\tan^{2}\left(m\psi_{\max}\right)}}=\frac{m}{\sqrt{4\tan^{2}\left(\psi_{\max}\right)+m^{2}}}=\frac{m}{\sqrt{4{\left(z_{\max}/z_{0}\right)}^{2}+m^{2}}},$$

we get the following expression for the SAM in the planes, where it achieves extreme magnitudes:

(32) $$\underset{r,\varphi }{\max}S_{z}\left(z=z_{\max}\right)=\frac{2}{W_{0}}{\left(\frac{a_{0}\sqrt{m}}{2w_{0}}\right)}^{m}\frac{e^{-m/2}}{\left[1+{\left(z_{\max}/z_{0}\right)}^{2}\right]{\left[1+{\left(2/m\right)}^{2}{\left(z_{\max}/z_{0}\right)}^{2}\right]}^{1/2}}.$$

This expression indicates that in each such plane with locally maximal SAM, the SAM achieves lower and lower magnitude, i.e., the strongest SAM is in the first plane given by Equations (29) and (30).

**Figure 4:**
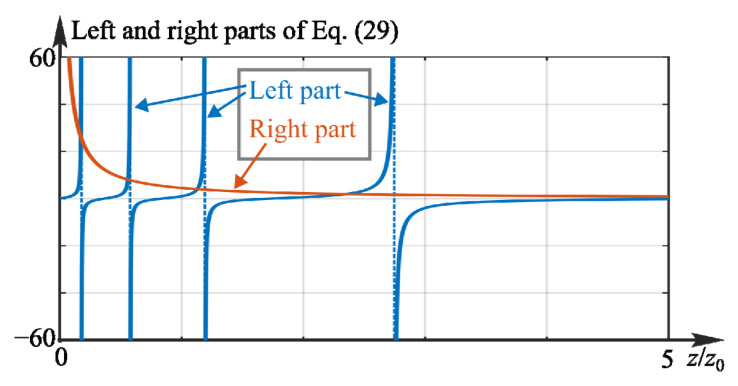
Left and right parts of Equation (29) as functions of *z*/*z*_0_ at *m* = 9.

The left and right parts of Equation (29) are shown in Figure 4. The right part is always positive and decays hyperbolically. The left part is a discontinuous function with the zeros in the points *z*_1,*p*_ = *z*_0_ tan(π*p*/*m*) (*p* = 0, 1, …) and with the discontinuities in the points *z*_2,*p*_ = *z*_0_ tan(π(2*p* + 1)/(2*m*)) (*p* = 0, 1, …). The roots of Equation (29) are thus in the intervals [*z*_1,*p*_, *z*_2,*p*_].

On the other hand, the left part of Equation (30) grows slower than the function tan(*mψ*) without the multiplier tan(*ψ*). Thus, the first root of Equation (30) should be between the values *ψ* = *m*^−1^arctan(*m*/2) and the discontinuity *ψ* = π/(2*m*).

Thus, the first root of Equation (29) is in the interval from *z* = *z*_0_tan[*m*^–1^arctan(*m*/2)] to *z* = *z*_0_ tan(π/(2*m*)). In our work, we will use the average value

(33) $$z_{\max}\approx \frac{z_{0}}{2}\tan\left[\frac{\arctan\left(m/2\right)}{m}\right]+\frac{z_{0}}{2}\tan\left[\frac{\pi }{2m}\right].$$

Section 7 below confirms that this is a good approximation at *m* > 3.

We note that the SAM magnitude is different for different values *a*_0_. In two extreme cases, when all the vortices merge in the center (*a*_0_ = 0) and when they move to infinity (*a*_0_ → ∞) the SAM should be equal to zero. In the first case, the light field reduces to an LG beam with *m*th-order cylindrical polarization which is not destroyed on propagation and the field has inhomogeneous linear polarization in an arbitrary transverse plane. In the second case, the superposition (5) consists only of the fundamental Gaussian beam while the portion of the LG beam with *m*th-order polarization vortex tends to zero. The Gaussian beam is linearly polarized and therefore the SAM should tend to zero.

To derive the radius of the polarization singularities that yield the maximal SAM, we should differentiate Equation (26) by *a*_0_. Thus, we get

(34) $$a_{0,\max}={\left(m!\right)}^{1/\left(2m\right)}\frac{w_{0}}{\sqrt{2}}.$$

At this value, the maximal SAM in an arbitrary transverse plane is then equal to

(35) $$\underset{r,\varphi }{\max}\;S_{z}\left(\alpha_{0}=\alpha_{0,\max}\right)=\frac{2}{\pi w_{0}^{2}}\frac{1}{{\left|q\right|}^{2}\sqrt{m!}}{\left(\frac{m}{2}\right)}^{m/2}\exp\left(-\frac{m}{2}\right)\sin\left(m\psi \right).$$

Applying the Stirling’s approximation *m*! ~ (2π*m*)^1/2^(*m*/*e*)*^m^* [26], we get

(36) $$\underset{r,\varphi }{\max}\;S_{z}\left(\alpha_{0}=\alpha_{0,\max}\right)\approx \frac{2}{\pi w_{0}^{2}}\frac{1}{{\left|q\right|}^{2}{\left(2\pi \right)}^{1/4}}\frac{1}{2^{m/2}m^{1/4}}\sin\left(m\psi \right)\leq \frac{2}{\pi w_{0}^{2}{\left(2\pi \right)}^{1/4}}\frac{1}{2^{m/2}m^{1/4}}.$$

This estimation indicates that the maximal achievable SAM decreases with increasing number of the polarization singularities.

We note that the linearly polarized Gaussian beam in the whole field has the following initial intensity distribution:

(37) $$I_{\mathrm{GB}}\left(r,\varphi ,0\right)=\frac{1}{W_{0}}{\left(\frac{a_{0}}{w_{0}}\right)}^{2m}\exp\left(-\frac{2r^{2}}{w_{0}^{2}}\right)$$

and its energy fraction in the whole energy is

(38) $$\begin{array}{l} W_{\mathrm{GB}}=2\pi \int_{0}^{\infty }I_{\mathrm{GB}}\left(r,\varphi ,0\right)rdr\\ \;\;\;\;=\frac{2\pi }{W_{0}}{\left(\frac{a_{0}}{w_{0}}\right)}^{2m}\int_{0}^{\infty }\exp\left(-\frac{2r^{2}}{w_{0}^{2}}\right)rdr={\left(\frac{a_{0}}{w_{0}}\right)}^{2m}{\left[\frac{m!}{2^{m}}+{\left(\frac{a_{0}}{w_{0}}\right)}^{2m}\right]}^{-1}. \end{array}$$

At the singularities circle radius from Equation (34) this energy reduces to

(39) $$W_{\mathrm{GB}}\left(a_{0}=a_{0,\max}\right)=\frac{1}{2}.$$

Thus, the maximal SAM density is achieved when the energy of the linearly polarized Gaussian beam in the superposition (5) is equal to the energy of the cylindrically polarized LG beam, i.e., equal to the half of the energy of the whole light field.

## 5. Orbital Angular Momentum Density

In paraxial light fields, only the longitudinal component of the OAM vector can be significant. It is equal to [27,28]:

(40) Jz=ImEx*∂Ex∂φ+Ey*∂Ey∂φ.

Substituting here the light field from Equation (5), we get

(41) Jz=1q2m+2w02mW0exp−2r2q2w02    ×Imrmcosmφ−a0mqm*∂∂φrmcosmφ−a0mqm+rmsinmφ*∂∂φrmsinmφ.

The second term is real and its imaginary part is zero. Then, the OAM density reduces to

(42) Jz=1q2m+2w02mW0exp−2r2q2w02Imrmcosmφ−a0mq*m−mrmsinmφ    =−mq2W0exp−2r2q2w02a0rqw02msinmψsinmφ.

It is seen that the OAM is equal to the SAM from Equation (22) but multiplied by −*m*/2.

It is in contrast with the conventional vortex beams with homogeneous circular polarization, whose OAM exceeds SAM *m* of −*m* times.

## 6. Analogy with Plane Wave and Revealing the Mechanism

According to Equation (5), the light field includes two opposite-charge circularly polarized LG beams. On propagation in free space, they rotate clockwise and counterclockwise. Thus, an angular analogue of standing wave is generated. This leads to a natural question whether the above-described effect can occur with the conventional standing wave if it is composed of two plane waves that have opposite tilt to the optical axis and opposite circular polarization. When superimposed with a plane wave without the tilt, such a field has the following complex amplitude:

(43) $$\begin{array}{l} E\left(x,y,z\right)=\frac{1}{\sqrt{W_{1}}}\exp\left(ik_{x}x+ik_{z}z\right)\left[\begin{array}{c} 1\\ -i \end{array}\right]+\frac{1}{\sqrt{W_{1}}}\exp\left(-ik_{x}x+ik_{z}z\right)\left[\begin{array}{c} 1\\ i \end{array}\right]\\ \;\;\;\;+\frac{a_{0}}{\sqrt{W_{1}}}\exp\left(ikz\right)\left[\begin{array}{c} 1\\ 0 \end{array}\right], \end{array}$$

where $k_{x}^{2}+k_{z}^{2}=k^{2}={\left(2\pi /\lambda \right)}^{2}$ and *k_x_* = sin α with α being the tilt angle. The multiplier *a*_0_ defines the relative strength of the plane wave without the tilt. The field (43) is of infinite energy, but to make the energy equal for different values *a*_0_, we introduced the multiplier $W_{1}^{-1/2}$ with $W_{1}=4+a_{0}^{2}$ (four scalar tilted plane waves and one wave without the tilt and with the amplitude proportional to *a*_0_).

It turns out that, on propagation in space, such a field also acquires nonzero SAM density:

(44) Szx,y,z=2ImEx*Ey=−4a04+a02sinkxxsink−kzz.

Due to infinite energy, plane waves do not spread on propagation. Therefore, the SAM density is repeated periodically and does not decay, in contrast to the realistic finite-energy beam (5), whose SAM density decays.

**Figure 5:**
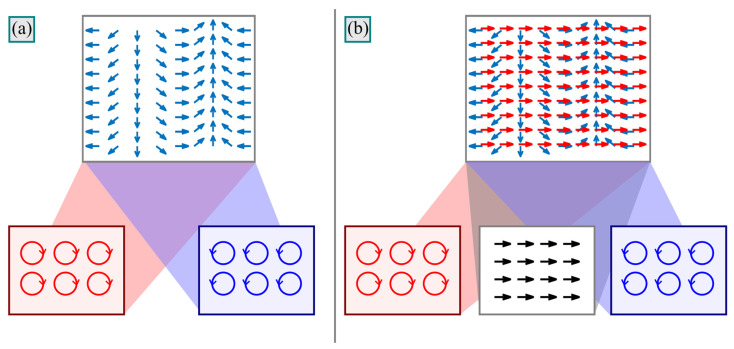
Generating a linearly polarized field in a superposition of two tilted plane waves with opposite tilts and with opposite circular polarizations (**a**), generating nonzero SAM density in a superposition of two tilted circularly polarized plane waves with a linearly polarized wave without the tilt:—tilted waves acquire phase retard and polarization becomes elliptic (**b**). Red and blue circles and black arrows in the initial fields denote respectively waves with right and left circular polarization as well as a linearly polarized wave. In the output distributions, blue and red arrows denote directions of linear polarization generated respectively by the circularly polarized waves and by the linearly polarized wave.

It is seen in Equation (44) that no matter how the beam without the tilt is polarized, the nonzero SAM would not occur without the difference *k* − *k_z_*. Figure 5 illustrates schematically interaction of two tilted plane waves with and without the non-tilted wave. The depicted area in Figure 5 has the size of 2λ × 2λ and computed for α = π/6. Thus, since *k_x_* = π/λ, the horizontal size of 2λ includes a single period over which the polarization direction rotates by an angle of 2π. Due to circular polarizations, electric vectors of the tilted plane waves rotate, but in opposite directions. These rotations cancel each other out and common polarization of tilted waves is inhomogeneous but remains linear (Figure 5a). The direction of linear polarization changes with the period of λ/sin α, which is decreasing with growing tilt. Adding linearly polarized non-tilted beam changes polarization direction, but leaves it linear in the initial plane, where all the waves are superimposed in phase. But on propagation, tilted waves become retarded compared to the non-tilted wave (Figure 5b). Thus, a phase delay appears between the tilted waves and non-tilted one. This delay means elliptic polarization.

However, if the electric field of the linearly polarized wave greatly exceeds or, vice versa, is much weaker than the electric field of tilted circularly polarized waves, then, despite the phase delay between them, elliptic polarization is close to linear and the SAM density is small. Thus, a question arises about the energies of the tilted waves and of the non-tilted wave that leads to the maximal SAM. Equation (44) follows that the maximal SAM magnitude is achieved at *a*_0_ = 2. This means that the energy of the linearly polarized wave is half of the energy of the whole superposition. Thus, we obtained just the same result as for the field (5) with multiple polarization singularities: the energy of the linearly polarized term should amount to half the energy of the whole field.

Elliptic polarization is not generated when the linear polarization of the two tilted circularly polarized plane waves is parallel to linear polarization of the non-tilted wave. Thus, according to Equation (44), if *k_x_x* = π*p* (*p* is an integer), then polarization is linear. On the contrary, when these vectors are orthogonal, polarization is closest to circular. This happen when cos(*k_x_x*) = 0. Thus, the maximal SAM magnitude of the field (5) should be achieved when ${\mathrm{LG}}_{m}\left(r,\varphi ,z\right)+{\mathrm{LG}}_{-m}\left(r,\varphi ,z\right)=0$, i.e., when cos(*mφ*) = 0. This occurs exactly at the above obtained polar angles *φ_p_* = (±π + 4π*p*)/(2*m*) (*p* = 0, …, *m* − 1).

The above explanation of the nonzero SAM also explains the proportionality between the OAM and SAM densities. It has no special physical meaning, but it is a consequence of the special-type complex amplitude (5). Indeed, the SAM density is due to the phase delay between the terms *r^m^*sin(*mφ*) and (*a*_0_*q*)*^m^*, whereas the OAM is contributed only by the *E_x_* component and it is due to the phase delay between the terms *r^m^*cos(*mφ*) and (*a*_0_*q*)*^m^*. It can be shown that for an arbitrary vector light field given by

(45) $$E\left(r,\varphi ,z\right)=\left[\begin{array}{l} A\left(r,\varphi \right)e^{i\Psi \left(r\right)}+B\left(r\right)e^{iΧ\left(r\right)}\\ \gamma \left(\partial A/\partial \varphi \right)e^{i\Psi \left(r\right)} \end{array}\right],$$

with *A*, *B*, *Ψ*, *Χ* being real functions and with *γ* being a real number, the SAM and the OAM densities are equal to

(46) $$\begin{array}{l} S_{z}=2\gamma B\left(\partial A/\partial \varphi \right)\sin\left(\Psi -Χ\right),\\ J_{z}=B\left(\partial A/\partial \varphi \right)\sin\left(\Psi -Χ\right). \end{array}$$

Thus, *J_z_* = *S_z_*/(2*γ*). For the field (5), *γ* = −*m*. That is why the OAM density in Equation (42) equals the SAM density (22) multiplied by (−*m*/2).

## 7. Simulation

**Figure 6:**
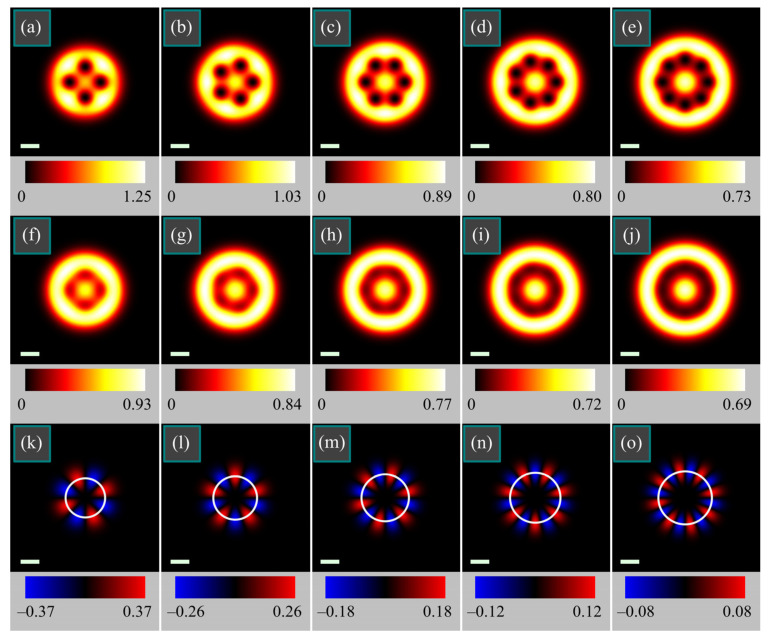
Intensity distributions in the initial plane (**a**–**e**) and at the maximal-SAM distance (33) (**f**–**j**), as well as SAM density distributions at the maximal-SAM distance (**k**–**o**) for the following parameters: wavelength λ = 532 nm, Gaussian beam waist radius *w*_0_ = 1 mm, number of the polarization singularities in the initial plane *m* = 4 (**a**,**f**,**k**), *m* = 5 (**b**,**g**,**l**), *m* = 6 (**c**,**h**,**m**), *m* = 7 (**d**,**i**,**n**), *m* = 8 (**e**,**j**,**o**), radius of the singularities circle *a*_0_ = 828 μm (**a**,**f**,**k**), *a*_0_ = 942 μm (**b**,**g**,**l**), *a*_0_ = 1041 μm (**c**,**h**,**m**), *a*_0_ = 1129 μm (**d**,**i**,**n**), *a*_0_ = 1210 μm (**e**,**j**,**o**), propagation distance is *z* = 0.349*z*_0_ (**a**,**f**,**k**), *z* = 0.284*z*_0_ (**b**,**g**,**l**), *z* = 0.240*z*_0_ (**c**,**h**,**m**), *z* = 0.208*z*_0_ (**d**,**i**,**n**), *z* = 0.183*z*_0_ (**e**,**j**,**o**). The radii *a*_0_ were computed by Equation (19) to equalize the intensities in the center and in the periphery. Circles on the SAM distributions (**k**–**o**) show the maximal-SAM radii obtained by Equation (25). All quantities (maximal intensity and SAM magnitude) are given in arbitrary units. Scale mark in each figure denotes 1 mm.

Figure 6 depicts the initial intensity distributions of the light field (5) for several orders *m* as well as the intensity and SAM density distributions on propagation in space to the plane with the maximal SAM. The singularities circle radius *a*_0_ was chosen so as to equalize the intensities in the center and in the periphery, i.e., by Equation (19). The intensity distributions were obtained by Equation (9), but were compared with those obtained by the numerical Fresnel transform implemented as a convolution with using the fast Fourier transform. All the figures looked identical. To make the magnitudes of the order of units, all field components were multiplied by a constant factor *C*_0_ = 3000.

Figure 6 confirms that the singularities circle radius computed by Equation (19) allows equalizing the beam intensity in the center and in the periphery. Figure 6 also confirms that in the transverse plane the maximal SAM magnitude is achieved at a circle of the radius given by Equation (25), which is √2 times smaller than the radius of maximal intensity of a single-ringed *m*th-order LG beam.

If is also seen in Figure 6 that the transverse plane with the maximal SAM is closer and closer to the initial plane with increasing number of singularities *m*. This is because this plane should be close than the first transverse plane with linear polarizations, which is also closer and closer, according to Equation (23).

**Figure 7:**
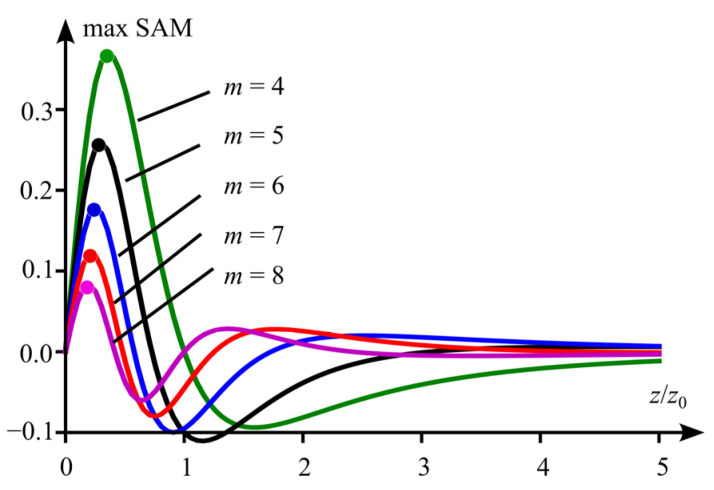
Maximal SAM for several numbers of the polarization singularities *m* when the singularities circle radius is chosen so as to equalize the intensities in the center and in the periphery. Dots in the top of each plot indicate the maximal-SAM distance obtained by the approximate Formula (33).

To verify the approximate expression (33) for finding the plane with the maximal SAM, Figure 7 illustrates the SAM dependence on the propagation distance for the beams shown in Figure 6. The SAM was computed by Equation (26). Dots in the top of each plot indicate the maximal-SAM distance obtained by Equation (33).

**Figure 8:**
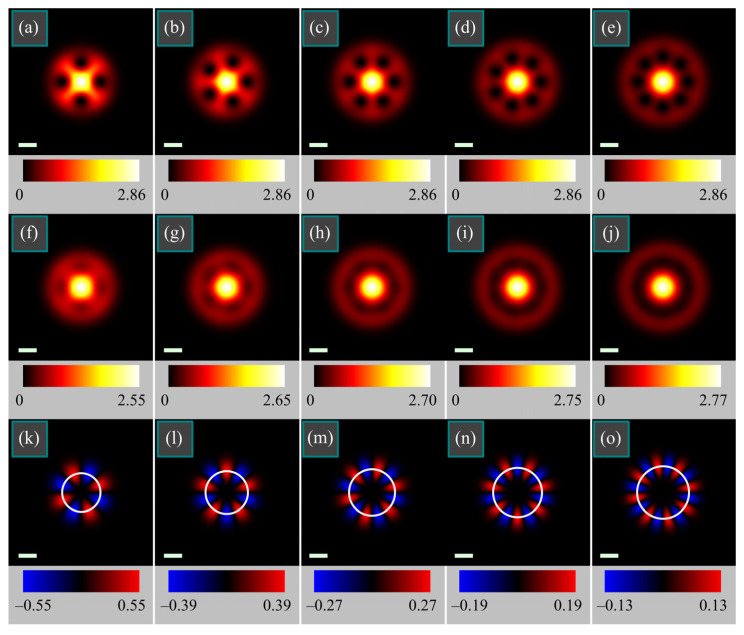
Intensity distributions in the initial plane (**a**–**e**) and at the maximal-SAM distance (33) (**f**–**j**), as well as SAM density distributions at the maximal-SAM distance (**k**–**o**) for the following parameters: wavelength λ = 532 nm, Gaussian beam waist radius *w*_0_ = 1 mm, number of the polarization singularities in the initial plane *m* = 4 (**a**,**f**,**k**), *m* = 5 (**b**,**g**,**l**), *m* = 6 (**c**,**h**,**m**), *m* = 7 (**d**,**i**,**n**), *m* = 8 (**e**,**j**,**o**), radius of the singularities circle *a*_0_ = 1052 μm (**a**,**f**,**k**), *a*_0_ = 1141 μm (**b**,**g**,**l**), *a*_0_ = 1223 μm (**c**,**h**,**m**), *a*_0_ = 1300 μm (**d**,**i**,**n**), *a*_0_ = 1371 μm (**e**,**j**,**o**), propagation distance is *z* = 0.349*z*_0_ (**a**,**f**,**k**), *z* = 0.284*z*_0_ (**b**,**g**,**l**), *z* = 0.240*z*_0_ (**c**,**h**,**m**), *z* = 0.208*z*_0_ (**d**,**i**,**n**), *z* = 0.183*z*_0_ (**e**,**j**,**o**). The radii *a*_0_ were computed by Equation (34) to maximize the SAM over all other radii *a*_0_. Propagation distances were computed by Equation (33) to maximize the SAM density. Circles on the SAM distributions (**k**–**o**) show the maximal-SAM radii obtained by Equation (25). All quantities (maximal intensity and SAM magnitude) are given in arbitrary units. Scale mark in each figure denotes 1 mm.

Figure 8 depicts the initial intensity distributions of the light field (5) as well as the intensity and SAM density distributions on propagation in space to the plane with the maximal SAM with all parameters being the same as in Figure 6, but the singularities circle radius is chosen so as to maximize the SAM density.

It is seen in Figure 8 that for each number of singularities *m*, the SAM density achieves magnitudes nearly 1.5 times higher than those in Figure 6.

In addition, it is seen that the initial fields have the same maximal intensity independently of *m*. This is because the maximal SAM is achieved, according to the above theory, when the energy of the Gaussian beam is equal to the half of whole beam energy. The rest energy of the same amount goes into the light ring and the intensity of this ring is weaker than the central intensity of the Gaussian beam. Thus, the central part of the intensity patterns of all the beams in Figure 8 is the Gaussian beam of the same energy, i.e., of the same amplitude. That is why the central intensity is the same.

**Figure 9:**
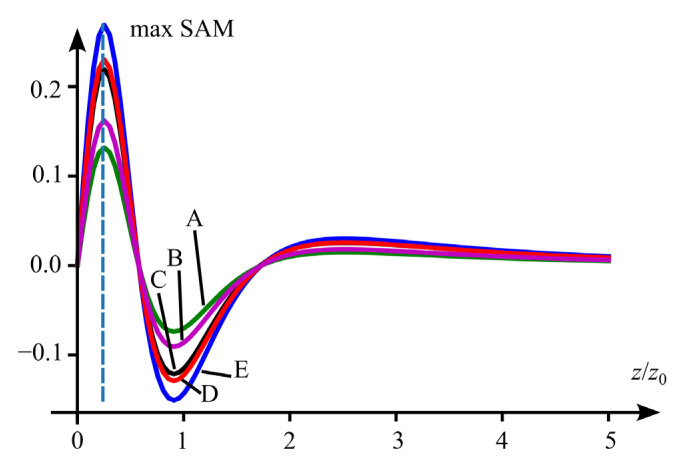
Maximal SAM of the light field (5) at *m* = 6 for several values of the singularities circle radius *a*_0_: *a*_0_ = *a*_0,max_ given by Equation (34) (curve E), *a*_0_ = 0.8*a*_0,max_ (curve A), *a*_0_ = 1.2*a*_0,max_ (curve B), *a*_0_ = 0.9*a*_0,max_ (curve C), *a*_0_ = 1.1*a*_0,max_ (curve D). Dashed line indicates the maximal-SAM distance obtained by the approximate Formula (33), which is independent on *a*_0_.

Now we verify that indeed the singularities ring radius *a*_0_ from Equation (34) yields the maximal SAM density over other radii. Figure 9 illustrates the longitudinal SAM distributions of the light field (5) at *a*_0_ given by Equation (34) and at some other values *a*_0_.

Figure 9 confirms that the maximal SAM is achieved at *a*_0_ given by Equation (34).

Finally, we compute the OAM density of the light field (5).

**Figure 10:**
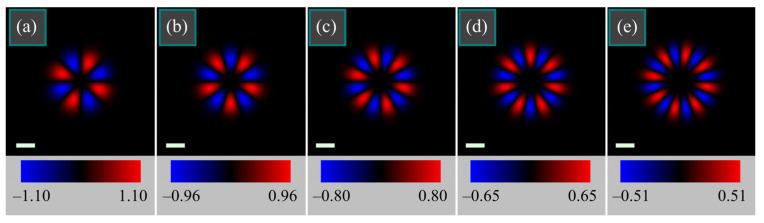
Distributions of the OAM density of the light field (5) at the maximal-SAM distance (33) for the following parameters: wavelength λ = 532 nm, Gaussian beam waist radius *w*_0_ = 1 mm, number of the polarization singularities in the initial plane *m* = 4 (**a**), *m* = 5 (**b**), *m* = 6 (**c**), *m* = 7 (**d**), *m* = 8 (**e**), radius of the singularities circle *a*_0_ = 1052 μm (**a**), *a*_0_ = 1141 μm (**b**), *a*_0_ = 1223 μm (**c**), *a*_0_ = 1300 μm (**d**), *a*_0_ = 1371 μm (**e**), propagation distance is *z* = 0.349*z*_0_ (**a**), *z* = 0.284*z*_0_ (**b**), *z* = 0.240*z*_0_ (**c**), *z* = 0.208*z*_0_ (**d**), *z* = 0.183*z*_0_ (**e**). The radii *a*_0_ were computed by Equation (34) to maximize the SAM (and thus the OAM) over all other radii *a*_0_. Propagation distances were computed by Equation (33) to maximize the SAM (OAM) density. All quantities are given in arbitrary units. Scale mark in each figure denotes 1 mm.

Figure 10 depicts the OAM density distributions of the light field shown in Figure 8. The OAM distributions look like inverted SAM distributions, but they were obtained by a quite different way: by Equation (40) where the angular derivative was represented as ∂/∂*φ* = *x*∂/∂*y* − *y*∂/∂*x* and was computed by using finite differences.

The maximal OAM magnitudes confirm that the OAM exceeds the SAM −*m*/2 times.

## 8. Experiment

In this section we experimentally show that, indeed, a superposition of *m*th-order and of 0th-order vector beams has 2*m* areas where polarization is elliptic and has different rotation direction. For the light beam we generated in the experiment, the Jones vector is given by

(47) $$E=\left(\begin{array}{l} \cos m\varphi \\ \sin m\varphi \end{array}\right)+\left(\begin{array}{l} a\\ 0 \end{array}\right).$$

**Figure 11:**
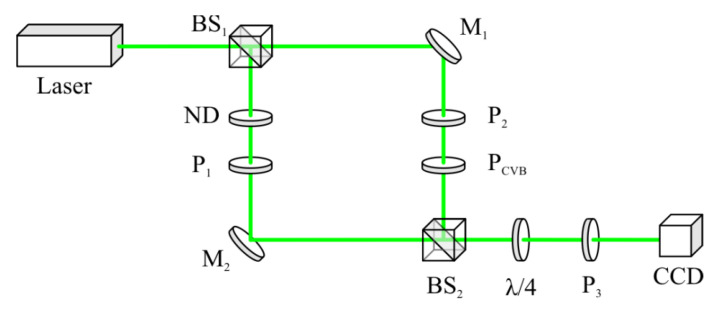
Optical setup. Laser is a laser MGL-F-532-700 (λ = 532 nm, 700 mW); BS_1_, BS_2_ are nonpolarizing beam splitter cubes; M_1_, M_2_ are mirrors; P_1_, P_2_, P_3_ are linear polarizers; ND is a neutral density filter; P_CVB_ is a vector wave plate (LBTEK VR1-532, Shenzhen, China), λ/4 is a quarter waveplate; CCD is a camera (ToupCam UCMOS10000KPA, Hangzhou, China).

Figure 11 shows the experimental setup. A linearly polarized light beam from a solid-state laser with a wavelength of 532 nm is divided into two identical beams after the splitting cube BS_1_. In one arm of the Mach–Zender interferometer, the beam passes through the first order q-plate (P_CVB_), which generates a cylindrical vector beam with *m* = 1. In the other arm of the interferometer, the amplitude of the beam with linear polarization is changed so that it is equal to *a* = 0.5. After the second splitting cube BS_2_, both beams are combined into one beam with the amplitude proportional to the beam (47).

In front of the registering camera, we placed a linear polarizer (P_3_) and a quarter-wave plate (λ/4) for measuring the components of the Stokes vector.

**Figure 12:**
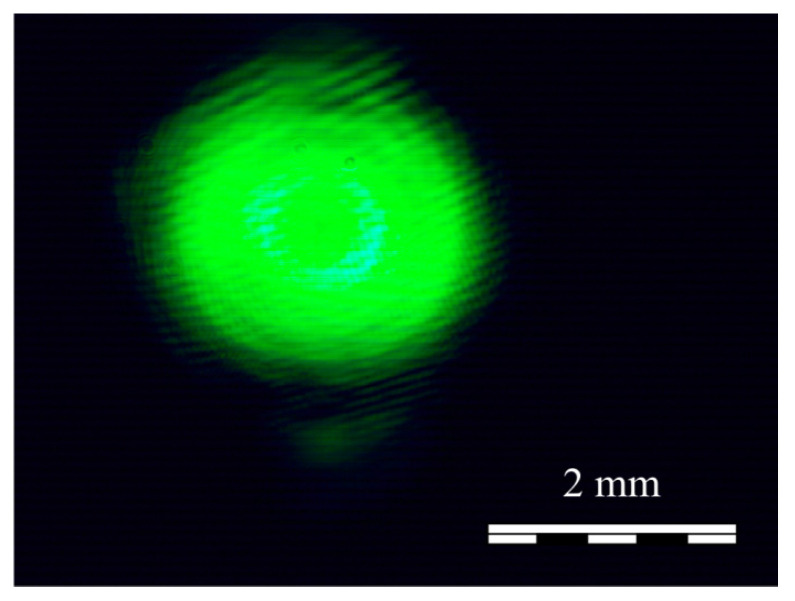
The intensity distribution of the beam from Equation (47) at *m* = 1 registered by the camera.

**Figure 13:**
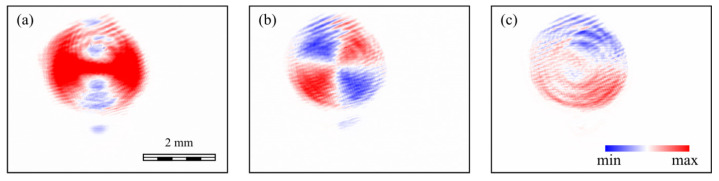
Measured components of the Stokes vector: s1 (**a**), s2 (**b**) and s3 (**c**). Blue and red colors denote the negative and positive values, respectively.

Figure 12 illustrates the beam intensity, registered by the CCD-camera without the polarizer P_3_ and quarter-wave plate in front of it, whereas Figure 13 depicts the components of the Stokes vector measured at different positions of the polarizer P_3_ and of the quarter-wave plate in front of the camera.

**Figure 14:**
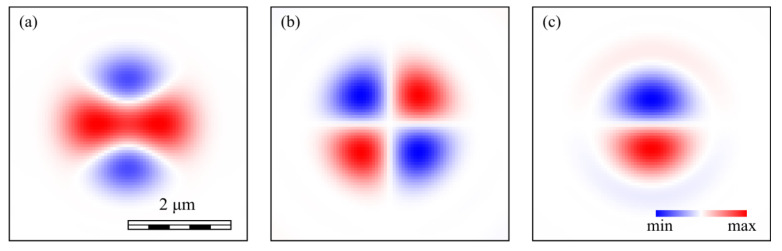
Components of the Stokes vector computed by the Richards-Wolf formulae: s1 (**a**), s2 (**b**) and s3 (**c**).

For comparison, Figure 14 shows results of a numerical simulation of focusing a light field with polarization (47) by using the Richards–Wolf formulae for the parameters *m* = 1 and *a* = 0.5 and for a lens with a low numerical aperture *NA* = 0.3.

Comparison of Figure 13 and Figure 14 indicates that in the beam from Equation (47) with the parameters *m* = 1 and *a* = 0.5, areas appear with elliptic polarization. These areas are observed both in the beam itself and when it is focused by a low-numerical-aperture lens.

## 9. Conclusions

We investigated paraxial light beams with multiple polarization singularities residing evenly on a circle (singularities circle). In the initial plane, polarization of such light beam is linear in all points, and the beam has zero spin and orbital angular momenta (SAM and OAM). When such a beam is propagating in free space, polarization is, in general, elliptic, and there are alternating areas with the positive and negative SAM, i.e., the spin Hall effect arises. In each transverse plane, the maximal SAM density magnitudes are achieved in 2*m* points (*m* points of maximal SAM density and *m* points of minimal SAM density) on a ring with a radius, equal to the half of the Gaussian beam radius multiplied by the square root from the number of singularities.

We obtained an approximate expression [Equation (33)] for the propagation distance where the SAM density achieves maximal magnitudes. This distance is independent of the singularities circle radius. We also derived an exact expression [Equation (34)] for the singularities circle radius that maximizes the SAM density. The maximal achievable SAM density is shown to decrease with the number of singularities *m*.

The investigated light beam is a superposition of a cylindrically polarized LG beam and of a linearly polarized Gaussian beam. We found that the maximal SAM density of the superposition is achieved when the energies of both beams are equal.

By considering an analogy with plane waves we found a reason of arising the spin Hall effect for the studied vector light field. It is due to different divergence of the cylindrically polarized LG beam and of the linearly polarized Gaussian beam.

We performed an experiment by generating a superposition of a linearly polarized beam with a cylindrical vector beam. This experiment confirmed that, indeed, 2m areas are generated where polarization is elliptic and has different rotation directions.

Application areas of the results obtained are designing micromachines for optical driving biological objects [29,30] or microtools in a lab-on-a-chip [31]. The SAM causes particles to rotate around their centers of mass [32] and engineering the SAM density distribution can allow simultaneous manipulating by an ensemble of particles. Another application is optical information transmission where the SAM density distribution can be used for encoding the data.

## Data Availability

Not applicable.
